# Effects of retained dead wood on predation pressure on herbivores in young pine forests

**DOI:** 10.1371/journal.pone.0273741

**Published:** 2022-09-06

**Authors:** Michelle Nordkvist, Stephanie Jonsson, Mats Jonsell, Maartje Johanna Klapwijk

**Affiliations:** Department of Ecology, Swedish University of Agricultural Sciences, Uppsala, Sweden; University of Saskatchewan College of Agriculture and Bioresources, CANADA

## Abstract

Retention of logging residue as dead wood could be a method to simultaneously increase biodiversity and predation rates of pest insects, in managed forests. Managed forests are generally low in diversity, and dead wood has been demonstrated to increase species diversity. Moreover, managed forests are predicted to suffer from higher frequency of insect outbreaks in the future, particularly in the northern hemisphere. In this study, we explore the effect of dead wood removal and addition in managed pine forest stands in Sweden on arthropod diversity and abundance and predation rates. We performed a controlled field experiment, focusing on logging residue type of dead wood. We used pitfall traps and sticky traps to measure arthropod diversity and abundance and plasticine larvae to assess predation rates. We specifically targeted generalist arthropods (i.e. non-wood living species), and predation rate on tree-dwelling larvae (corresponding to defoliating outbreak pests). We found no effect of dead wood addition on arthropod abundance or diversity, neither did we find an effect on predation rate. Despite the lack of effects in our study, we argue that dead wood can be an important component for both biodiversity of generalist arthropod and for pest control, but the effect may depend on both the specific arthropod group targeted and the specific life stage of the pest insect as well as on inherent components of the dead wood, such as age.

## Introduction

Dead wood is an important resource for organisms in forest ecosystems [1]. Besides acting as breeding substrate for many forest species [2] it is an important structural component, providing for shelter and altering micro-climate [3]. Dead wood may also affect top-down regulation of herbivores through effects on their natural enemies [4].

The amount of dead wood in managed forests (within this context plantation forest) is generally low due to forestry practices [5–7] and the increased use of bioenergy from forest biomass to substitute fossil fuels, could lead to further decrease in dead wood availability [8]. Logging residues (i.e. tops, twigs and branches) are used to substitute fossil fuels and therefore increased removal can be expected [9]. Especially in production forests the retention of logging residues may be important for the increased provision of structural diversity through the creation of more microhabitats.

Removing logging residues can have negative effects on biodiversity [10,11] especially for saproxylic (wood living) arthropods [8]. Less is known about the value of dead wood for non-wood living arthropod taxa. The response of these groups to removal or retention of dead wood is likely more heterogeneous, as variation between studies is large [12]. The response appears to depend on how respective species are associated with microclimatic variables such as sun exposure, soil moisture and climate [13,14]. Tree species, diameter, decay stage etc. of the dead wood could also be hypothesized to influence the response, in the same as they do for saproxylic species [12,14,15]. Hence, retaining logging residues could be beneficial for diversity and abundance of both saproxylic [12,16] and non-saproxylic arthropods [12,14]. Studies exploring the effect of dead wood on non-saproxylic taxa, particularly at later stage of wood decay, are lacking [12].

In addition, forest heterogeneity and complexity created by dead wood could alter ecological processes such as pest suppression [4]. Higher predation rates of European pine sawfly cocoons were found when dead wood was present in monocultures [4], potentially by providing shelter to small mammalian predators [17]. Cocoon predation is an important regulating factor for sawfly populations [18] and is thought to be one of the regulation factor to reduce the probability of outbreaks. Similarly, dead wood could be beneficiary for generalist arthropod predators [19,20], such as ants and spiders, important enemies to the larval stage of pest insects such as the pine sawfly [21–24]. A high level of heterogeneity provides alternative food resources and alternative shelters which could lead to higher abundance and/or diversity of predators, potentially increasing top-down mortality of insect herbivores. A study by Poch & Simonetti [25] demonstrated increased predation rates of tree-dwelling larvae in *Pinus radiata* stands with higher understory complexity. We are not aware of any studies assessing the effect of dead wood on herbivorous larval predation rates.

Production forests are thought to be more susceptible to damage by forest insects due to their structural simplicity [26,27] and pest insect outbreaks are expected to increase in the future, particularly in the northern hemisphere mainly due to climate warming [28]. Increased probability and magnitude of insect pest damage is a threat to e.g. wood biomass production, creating the need to increase the resilience of production forests to disturbances. Dead wood retention could contribute to increased predation pressure of potential pest insects, and reduce the risk for outbreaks.

We set out to explore the effect of dead wood addition and removal on the abundance and diversity of arthropod predators and investigate whether it might have an effect on the predation of pest insects. We conducted our study in monoculture production stands of pine, in which dead wood had either been added or removed in 2015. We assessed the diversity and abundance of ground dwelling arthropods in these stands, and focused specifically on attention to the diversity and abundance of predators. Further, we used plasticine model larvae to assess attack rates (a proxy of predation pressure) on pine trees, and to test if predation is related to dead wood occurrence. We used sticky traps on half the trees to quantify the contribution of ground-dwelling predators to these attack rates.

We set out to answer the following research questions:

1. Does the presence of dead wood affect the abundance and diversity of ground-dwelling arthropods?
2. Does dead wood manipulation affect the abundance and diversity of ground-dwelling predators?
3. Does the presence of dead wood affect predation pressure of folivorous insect larvae and is this related to the abundance and diversity of ground-dwelling arthropod predators?

We hypothesised that arthropod abundance and diversity would be greater in the presence of dead wood [12,14] and that that would result in higher predation pressure on herbivorous larvae. The novelty of this study lies in the attempt to simultaneously study the effect of dead wood on species communities and pest control.

## Materials and methods

### Study area

This study was conducted in 2020, in five monoculture Scots pine (*Pinus sylvestris* L.) stands (from here on referred to as sites) located between Månkarbo (60.2268° N, 17.4646° E) and Björklinge (60.0318° N, 17.5521° E) in the Uppland region of south central Sweden (S1 Table). Planted seminatural coniferous forest dominates in the region and clear felling is the dominant management regime. The sites were between thirteen and 20 years old and prior to pre-commercial thinning.

### Experimental design

The experiment was initially set up in 2015 to study the effect of dead wood addition and removal on sawfly cocoon predation [4]. Ten trees per site were selected using the following method: A 150×90 m grid with fifteen equally sized cells was placed over each site whereby ten cells were randomly selected. Within each cell, the tree closest to the centre of the cell was chosen and defined as the sample plot. This resulted in 50 plots (ten per site). At each plot, dead wood was either added or removed through a randomized selection process. We refer for further specifics of the methodology to Bellone *et al*. 2017 [4].

**Fig 1:**
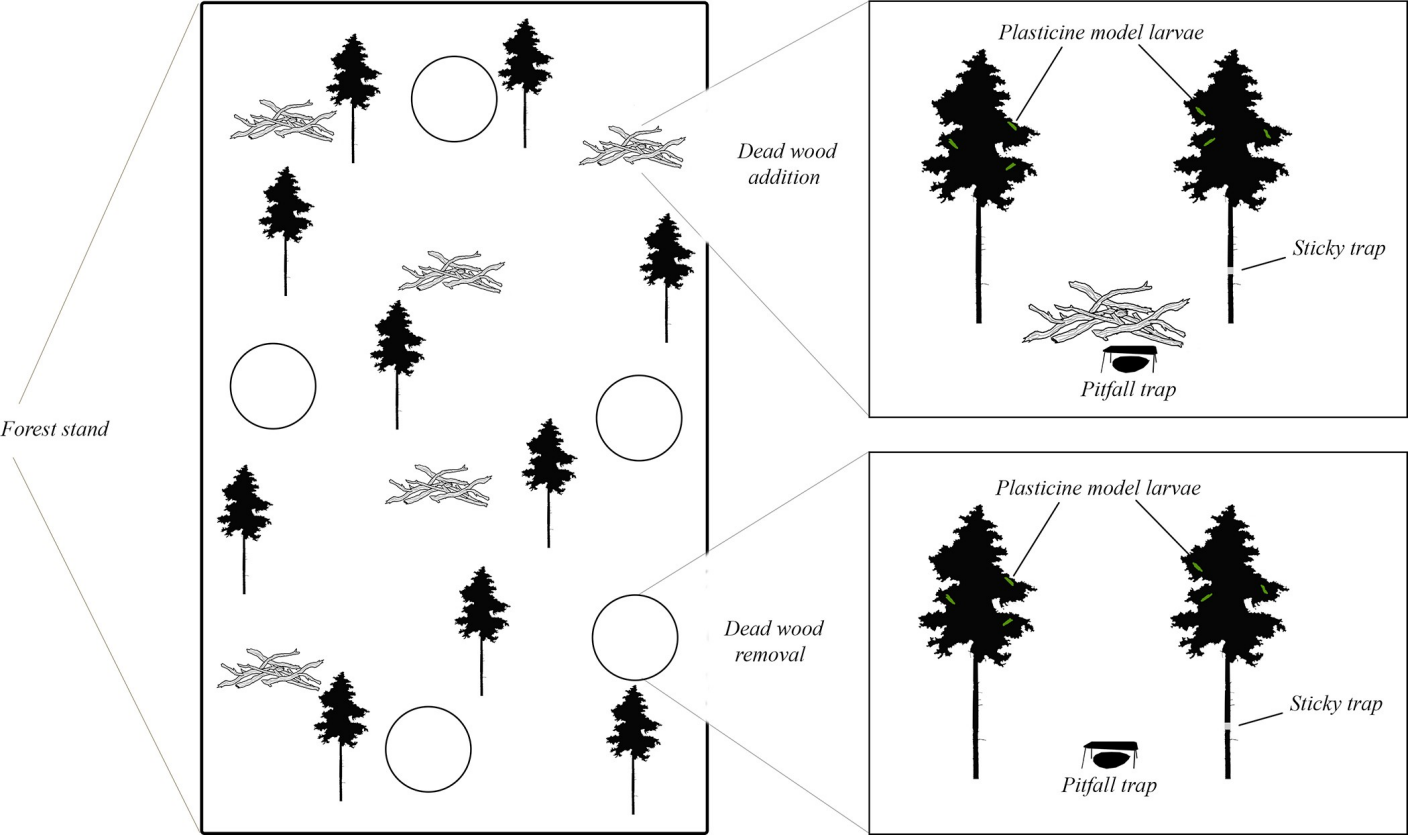
Experimental design testing the effect of dead wood addition/removal on arthropod abundance and diversity as well as predation pressure on plasticine larvae models. Each forest site had five plots where dead wood had been added (pile of branches) and five plots where dead wood had been removed (empty circles). At each plot, three different methods were used to collect data: Pitfall traps, plasticine model larvae and sticky traps. Each plot had two experimental pine trees were attack rates were measured by using plasticine larvae models attached on the tree branches. Sticky traps were attached on one of the two experimental trees at each plot to estimate the effect on attacks when the number of predators walking up the stem were reduced. One pitfall trap was placed in each plot.

In 2020, we re-used the existing experimental set-up. At each plot, a second tree was selected creating a set up with two trees at each plot, i.e. in total 100 trees (20 per site). The second tree, of similar size, was randomly selected within a 4 m radius from the first tree. Plots with added dead wood had the dead wood pile between the two selected trees. In total the experiment contained 25 replicates where dead wood was added and 25 replicates where dead wood was removed, equally divided over the five sites (Fig 1).

### Data collection

We conducted the field experiment during four weeks, from 15^th^ of May to 14^th^ of June 2020. Ground-dwelling arthropods were sampled using pitfall traps. Sticky traps were used to quantify the number of walking predators on tree stems. Attack rates on herbivores were measured using plasticine model larvae placed on the chosen pine trees.

#### Pitfall trapping

One pitfall trap, consisting of a plastic jar with a diameter of 11.5 cm and depth of 12 cm, was placed at each plot, resulting in 50 traps in total. The traps were covered with a plastic roof suspended 5-10cm above ground-level. The collection jar of each trap contained 150ml of 50% propylene glycol used as killing and preservation fluid. In plots where dead wood had been added, traps were placed near the largest pile of dead wood (on average 0.6 m from the centre of the pile, standard deviation 0.15), while in plots where dead wood was removed they were placed between the two experimental trees. The traps were active during all four weeks of the experiment. One trap was damaged and excluded from the analysis. All sampled arthropods were sorted to order level. Hymenopterans, Coleopterans and Araneae were further sorted to family level to better separate predators from other feeding groups. Insect larvae were not identified and therefore excluded in the analysis. We counted the total number of arthropods, predators, ants, ground beetles and spiders caught in the pitfall traps. Individuals belonging to Formicidae, Carabidae and hunting spider families (Gnaphosidae, Lycosidae, Pisauridae, Salticidae, Thomisdae and Zoridae) were defined as predators. Since we categorised predators on group level, we decided to define predators as groups in which a majority of species are predatory. Moreover, we aimed to select groups which we know are important predators for tree-dwelling pest insects; spiders and ants [23,24].

#### Plasticine model larvae

Attack rates on herbivore larvae were quantified using plasticine model larvae. Plasticine larvae are artificial prey made from clay (smeethi standard green) that resembles real prey [29]. It has been shown to be a reliable method to assess relative differences in predation rates between experimental treatments [25,30,31]. In the middle part of all experimental trees (i.e., 6^th^ or 7^th^ whorl, about 1.8 m above ground), three plasticine model larvae were placed on the 2^nd^ of June, and re-collected after one week. Plasticine models were formed around a copper wire (sticking out in both ends). The wire was used to attach the models on the tree branches. Pictures of every model were taken right after placement and before re-collecting the model, reducing the risk of including marks created while placing or collecting the models. Detection of marks made from predators on the plasticine larvae was done with a magnifier and compared to photographs from before and after the placement as a control reference. The number of attacked larvae within each tree was counted (maximum three). Since we were only interested in attacks from arthropods, we excluded six model larvae that had been attacked only by birds. Whether marks were done by birds or arthropods was determined using [32].

#### Sticky traps

Sticky traps were placed on one of the two trees at each lot about one meter above the ground. We used odourless sticky traps with a width of 5 cm, specifically developed for catching insects on tree stems (Swissinno Solutions). The traps were active the two last weeks of the experiment (29^th^ of May to 14^th^ of June). They were used for two purposes. 1) To quantify the number and identity of predators walking up the tree stems and 2) to exclude walking predators on one of the experimental trees to be able to relate the pitfall trap catches to predation rates. All ants and spiders were counted. We only counted spiders and ants, since our interest was in walking predators and because these groups were abundant (both in the pitfall traps and on the sticky traps).

### Statistical analyses

#### Pitfall trap data

R software version 3.6.3 was used for all statistical analyses [33]. To test the effect of dead wood addition/removal on the following five response variables: total arthropod abundance, predator abundance, hunting spider abundance, ground beetle abundance and ant abundance, generalized linear mixed models with negative binomial distribution was fitted. We used negative binomial distribution and not Poisson distribution since overdispersion was detected when inspecting the residuals (dispersion_glmer; blmeco package; [34]). To test the effect of dead wood addition/removal on diversity, number of unique orders, predator groups as well as families within the orders of Aranaeae and Coleoptera were counted, per sample. Number of orders, number of predator groups, number of Coleopteran families and number of Araneae families were used as measures of diversity, and to test the effect of dead wood generalised linear mixed models with negative binomial distribution were fitted. Additionally, we performed individual based rarefaction analysis to assess the difference in order, beetle family and spider family numbers. In all models, dead wood addition/removal was set a fixed effect and site as a random effect (glmer.nb(); lme4 package; [35]). Significance was tested with an Anova type II Wald chi-square test using the Anova() function in the car package [36]. Homogeneity of variances were tested by using Levene’s test, using the Levenetest() function in the car package [36]. Rarefaction was performed using the iNEXT package [37]. In the rarefaction analyses, if the curves (dead wood added vs. removed) were clearly situated outside each other’s confidence limits at the point of comparison they were deemed as significantly different [38].

#### Plasticine larvae and sticky trap data

To test whether the proportion of attacked larvae was sinificantly related to the presence of dead wood, a generalized linear mixed model with binomial distribution was fitted. Dead wood addition/removal and presence/absence of sticky traps and their interaction were set as a fixed effects and plot nested in site as a random effect. Since the interaction was not significant it was removed from the model (model output with interaction presented in the S1 and S5 Tables), and only dead wood treatment and sticky traps was used as fixed factors. The glmer() function in the lme4 package was used [35]. Significance was tested with an Anova type II Wald chi-square test (Anova() function, car package; [36]).To test if ant and spider catch on sticky traps differed between the dead wood treatment a generalized linear mixed model with negative binomial distribution was fitted. Dead wood addition/removal was set as a fixed effect and site as a random effect. The glmer.nb function in the lme4 package was used [35], and significance was tested with an Anova type II Wald chi-square test (Anova() function, car package; [36]). Correlations between total arthropod catch and attack rates, and between total predator catch and attack rates was done with a Pearson correlation test (R stats package; [33]).

## Results

### Arthropod diversity and abundance (pitfall traps)

In total we caught 15 516 arthropods belonging to sixteen identified orders in the pitfall traps. Six orders made up 98% of the specimens: Hymenoptera (4902; out of which 4777 belonged to the family Formicidae), Acari (2929), Araneae (2769), Collembola (2641), Diptera (1040) and Coleoptera (970). Ten additional orders were represented by the remaining 259 specimens (full list provided in S2 Table). Insect larvae as well as twelve unidentified specimen were excluded from the analysis. Out of the total sample, 6313 specimen could be classed as predators, being either ants, ground beetles or hunting spiders.

**Fig 2:**
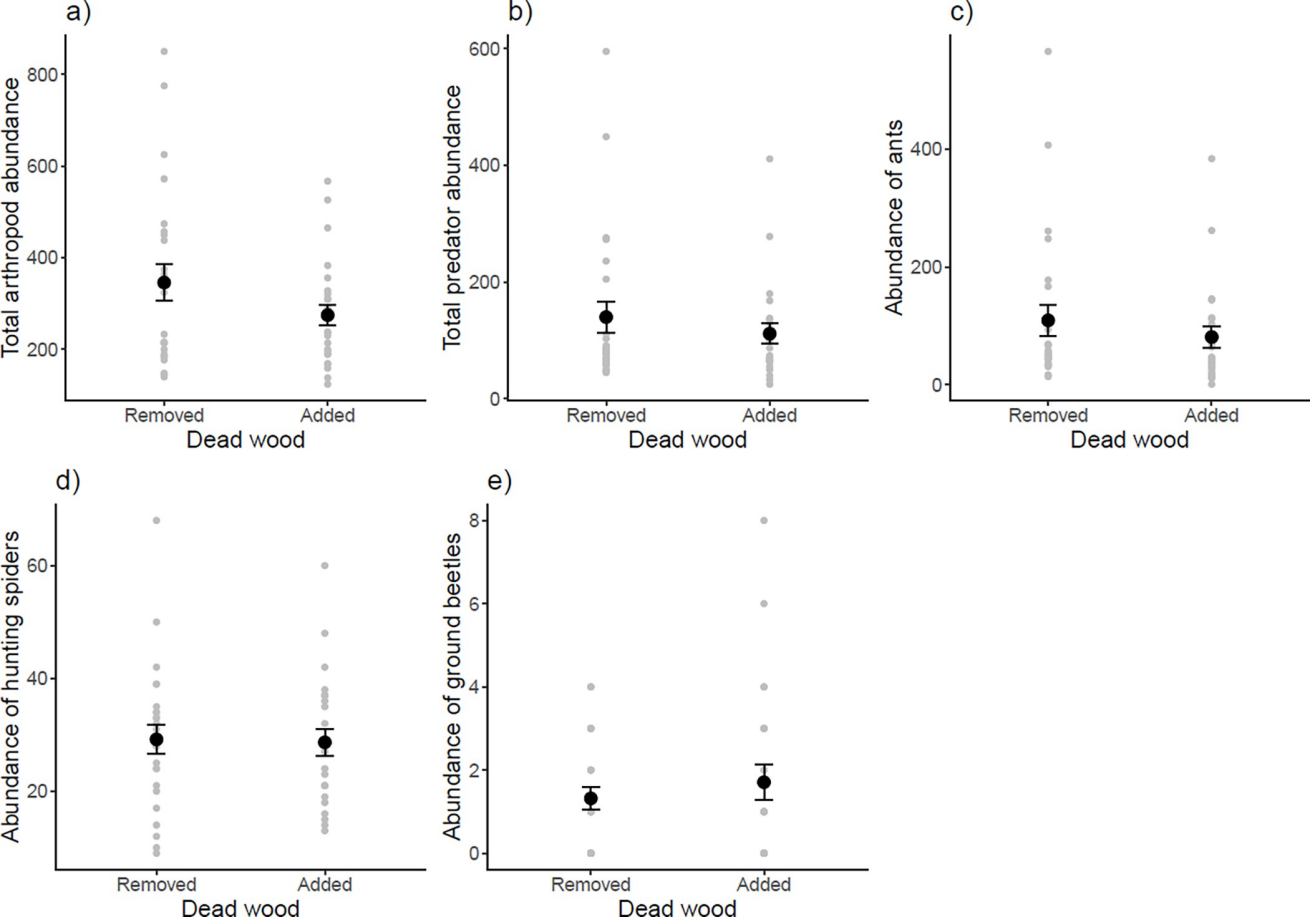
Abundance of a) all arthropods, b) all predators, c) ants, d) hunting spiders and e) ground beetles caught in pitfall traps in relation to dead wood treatment; removed or added. Grey points represent the raw data points, i.e. the number of individuals in one sample. Black points and error bars represent the mean value and standard error. Mean (±SE): 346±39, 275±23, 140±27, 112±17, 109±27, 81±18, 29±2.6, 29±2.4, 1.3±0.27 and 1.71±0.43 from top to bottom and left to right.

For abundances, there were no differences between plots with removal or addition of dead wood for any of our response variables (total arthropods, predators, ants, hunting spiders and ground beetles, S3 Table, Fig 2). Although not significant, total number of arthropods, predators and ants contradicted our hypothesis, as there were fewer of those where dead wood had been added (S3 Table, Fig 2).For total arthropod number this pattern was significant on the 0.1 level (p = 0.06, χ^2^ = 3.46, df = 1).

**Fig 3:**
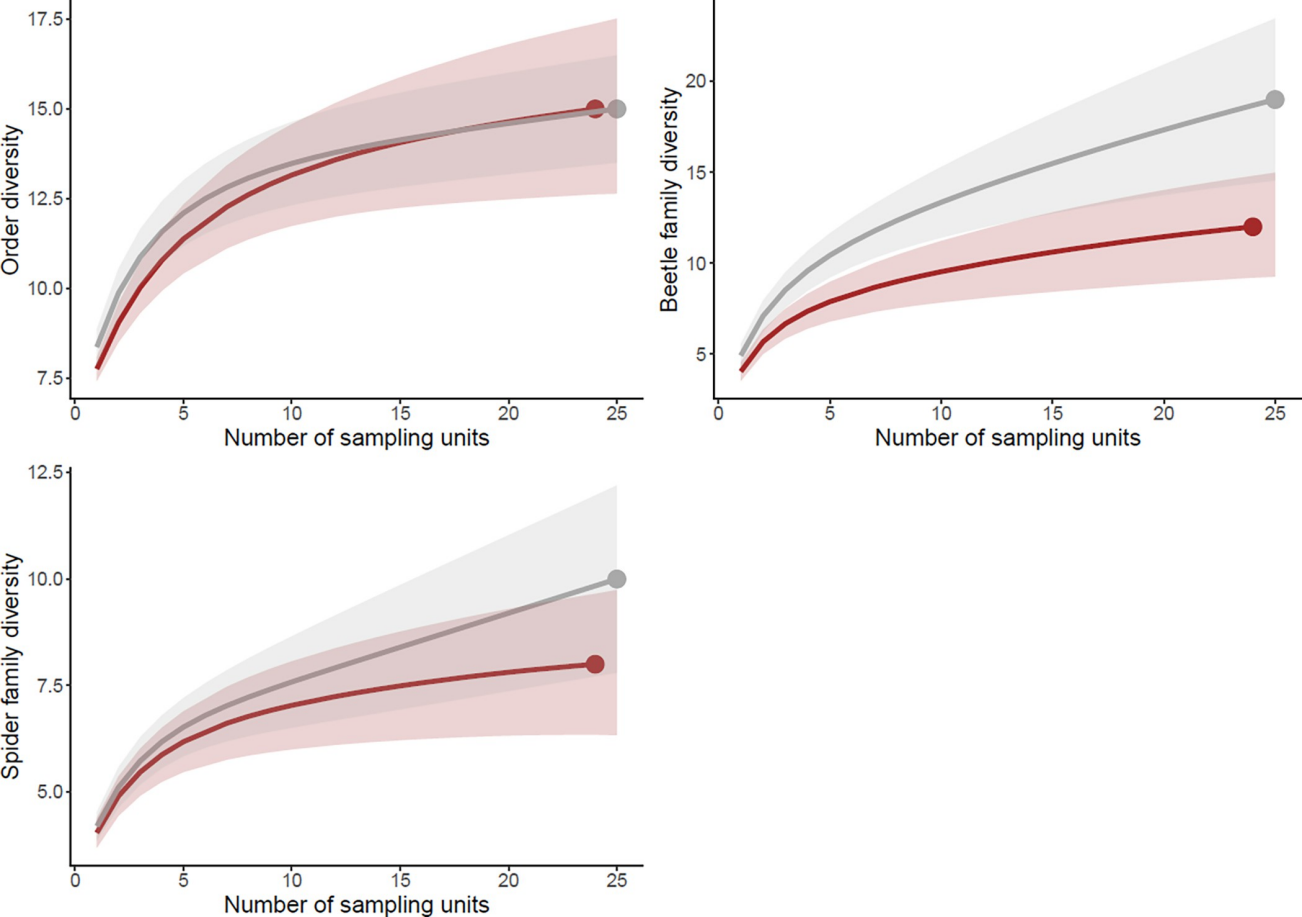
Rarefaction curves for number of orders, beetle families and spider families (left to right, top to bottom) for the two dead wood treatments; removed (grey) and added (red). Shaded area represent the 95% confidence interval and the end point is the observed number of taxa.

Regarding the number of taxa; order and spider family numbers did not differ between plots with dead wood addition and removal (S4 Table). Beetle family numbers showed a tendency to be higher where dead wood had been removed when analysed per plot (alpha diversity) (p = 0.15, χ^2^ = 2.0, df = 1; S4 Table). The total number of beetle families summed for all plots (gamma diversity) was higher where dead wood was removed than where it was retained (Table 1, Fig 3). For spider family number the difference was similar but not significant, whereas for number of orders there was no difference between treatments (Table 1, Fig 3).

**Table 1:** Rarefaction analyses output. The number of observed taxa with 95% confidence limit, for number of orders, beetle families and spider families when dead wood was either added or removed.

Number of species with 95% confidence limits		
	**Dead wood removed (n = 25)**	**Dead wood added (n = 24)**
Number of orders	15±1.5	15±2.4
Number of beetle families	19±4.5	12±2.8
Number of spider families	10±2.2	8±1.7

### Attack rates and abundance of ants and spiders on sticky traps

**Fig 4:**
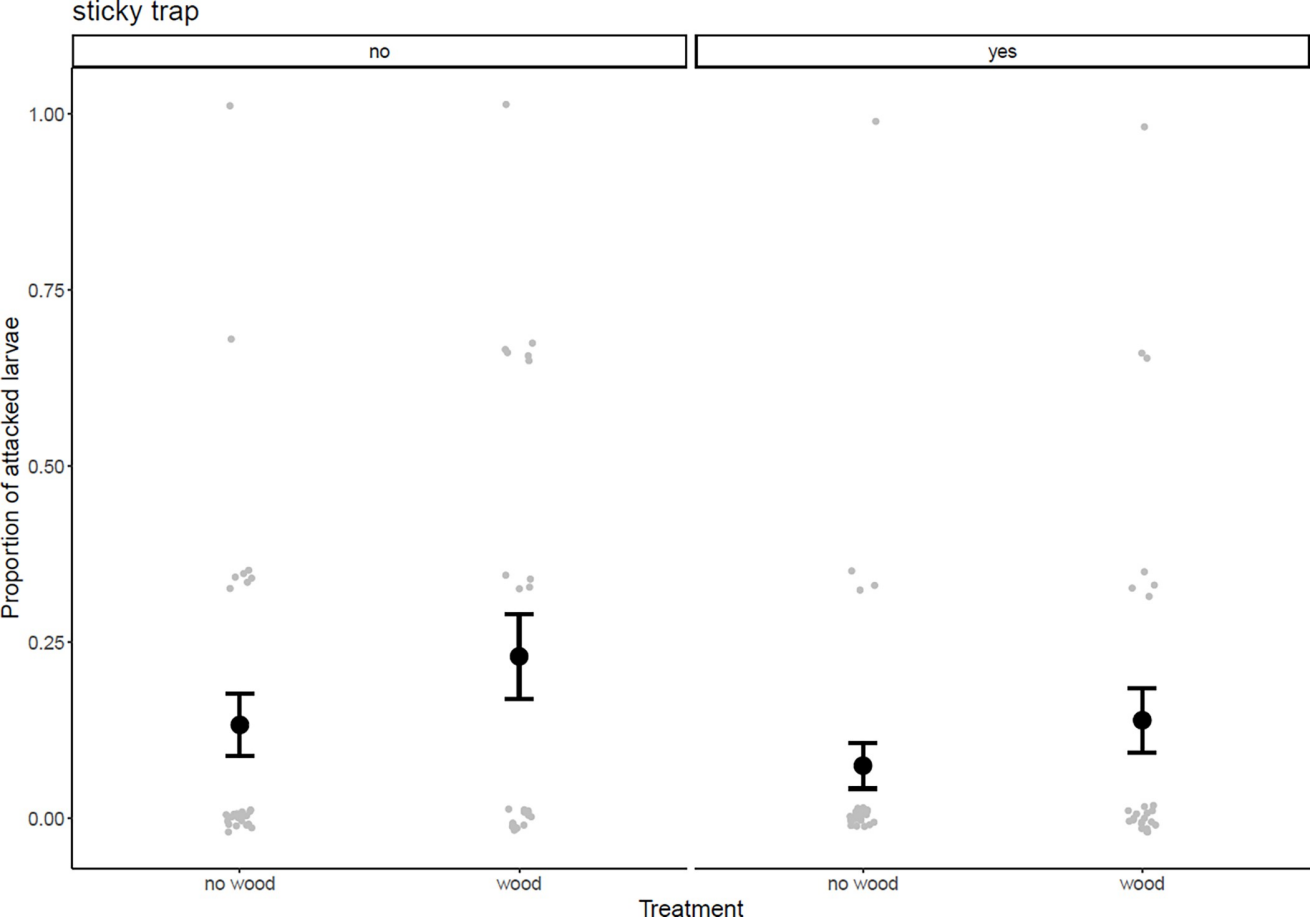
The proportion of attacked plasticine larvae on trees without and with sticky traps (no/yes) in relation to dead wood treatment; removed or added. Grey points represent the individual data points (i.e the proportion of attacked larvae per tree, 0, 1, 2, or 3 out of 3) and black points and error bars represent the mean value and standard error. Mean±SE: 0.14±0.05, 0.24±0.07, 0.08±0.04 and 0.15±0.05, from left to right.

Of the total 300 plasticine model larvae, 45 models showed attack marks from arthropods. There was no significant difference in attack rates on plasticine model larvae between plots with added and removed dead wood nor was there an interaction (Table 2, Fig 4). Trees with sticky traps had lower attack rates compared to trees without sticky traps, 11 and 19% respectively (p = 0.048, χ^2^ = 3.91, df = 1; Table 2, Fig 4). Due to the relatively high number of zeros (no attack) the results should be interpreted with caution. The lack of an effect on attack rates is however consistent with the null effect of dead wood on arthropod predators.

**Table 2:** Anova (type II test) and summary table for generalised linear mixed models testing the difference in proportion of attacks against the dead wood treatment (added or removed). Dead wood treatment and sticky traps were used as a fixed factors, site and plot nested in site as a random factors. Significant effects are marked in bold.

Larvae attacks (proportion)					
**Fixed**	Estimates	SE	χ^2^	df	p-value
Intercept	-2.32	0.46			<0.001
Treatment			2.44	1	0.12
Wood (added)	0.82	0.53			
Sticky trap			3.91	1	**0.048**
Sticky trap (yes)	-0.73	0.37			
**Random**	Variance	Standard dev.			
Site	0.006	0.08			
Site/Plot	1.5	1.23			

On the sticky traps we counted 527 ants and 195 spiders in total. There was no difference in number of ants caught on the sticky traps between plots with removal or addition of dead wood. There was no significant difference in number of spiders either, however a trend towards higher number of spiders on sticky traps in plots where dead wood was added (p = 0.12, χ^2^ = 2.36, df = 1) (Table 3). Mean (±SE) number of spiders caught on trees in plots with added and removed dead wood was 3.2±0.73 and 4.6±0.96, respectively.

**Table 3:** Anova (type II test) and summary table for generalised linear mixed models testing the difference in number of a) ants and b) spiders caught on the sticky traps against the dead wood treatment (added or removed). Dead wood treatment was used as a fixed factor, and site as a random factor.

**(a) Ants on sticky traps**					
**Fixed**	Estimates	SE	χ^2^	df	p-value
Intercept	2.01	0.42			< 0.001
Treatment			0.01	1	0.92
Wood (added)	0.035	0.34			
**Random**	Variance	Standard dev.			
Site	0.62	0.79			
**(b) Spiders on sticky traps**					
**Fixed**	Estimates	SE	χ^2^	df	p-value
Intercept	1.08	0.24			< 0.001
Treatment			2.36	1	0.12
Wood (added)	0.36	0.24			
**Random**	Variance	Standard dev.			
Site	0.15	0.38			

There was no correlation between attack rates and number of caught arthropods or predators in the pitfall traps (Table 4).

**Table 4:** Summary table for a Pearson correlation test between the number of attacked plasticine larvae and (a) predator abundance and (b) total arthropod abundance caught in the pitfall traps.

**(a) Correlation between predator abundance and attacks**			
Cor	t	df	p-value
0.08	0.53	48	0.6
**(b) Correlation between total arthropod abundance and attacks**			
Cor	t	df	p-value
-0.1	-0.67	48	0.5

## Discussion

Contrary to our expectation, we found no strong effects of adding or removing dead wood on arthropod abundance or diversity, neither in the pitfall traps nor on the sticky traps. Similarly, we did not find an effect of the presence or absence of dead wood on attack rates (predation).

### Arthropod abundance and diversity

One of our main expectations was that the addition of dead wood would increase arthropod diversity and abundance due to increased structural complexity [12,13,39,40]. Instead, we found no strong effects on either abundance or diversity. A recent review showed a varied response of non-saproxylic arthropods to dead wood presence [12]. Both neutral [41] and negative [13] effects have been demonstrated. Moreover, when dead wood is removed a more sun exposed (warmer and drier) microclimate should be created, potentially favouring more taxa [15] or increasing arthropod activity. Thus, if certain species groups respond negatively to dead wood, and other groups positively [12,15], this could result in a close to net zero effect on total abundances at the coarser taxonomic level used in this study. When comparing the catches on sticky traps, spider abundance showed a trend to a positive response to dead wood. Our results follow the previous finding by [40] that spiders tend to increase in abundance in relation to increased structural complexity.

Similarly, the structural complexity of dead wood of importance for shelter and microclimate, decreases during succession: first foliage falls off, then the finest twigs and later coarser twigs are lost. We know that for saproxylic species, wood in later decay stages is often more species rich [38], but this may not be the case for non-saproxylic organisms. For example, abundance of Carabids responds positively to fresh dead wood [13,41] but abundances are lower when associated with older dead wood [15], suggesting for this coleoptera group that logging residue may be of higher value as a structural component in earlier stages of decomposition.

The high nutrient content of the fresh wood may also indirectly attract predators. High supply of nutrients could be beneficial for prey, probably mainly detrivores attracted to the mouldy environment, increasing density of available prey. The dead wood in our study was at least five years old (piles created in 2015; [4]) and during the succession of dead wood, the nutrient content of the wood decreases as it is consumed. Moreover, it has been shown that positive effects of dead wood on non-saproxylic arthropods could be mediated via changes in sun exposure [12]. Dead wood may be more important in a sun exposed environment as it may mitigate microclimate extremes. On a fresh clear cut sun exposure is high, and then diminishes over time as the vegetation grows. These processes might lead to that the initial positive effect become a neutral or negative effect, at least on certain species groups, after some years. Our results therefore suggest that a temporal trend in succession of dead wood is reflected also in non-saproxylic species communities. We believe that experimental studies targeting questions such as how arthropod abundance and richness is affected by harvest residue over time. This could be done by conducting an experiment, similar to the one in this study, in a clear cut with harvest residue and repeating it over a number of years.

One caveat that needs to be mentioned as it might have influenced our results, is the differing amounts of (non-experimental) dead wood at stand level. [42] showed a strong positive relationship between the number of captured arthropods and increasing volume of dead wood at stand level, though no consistent relationships were found when they looked at arthropod captures in proximity to piles of dead wood within the same stands. Hence, they suggested that with high volumes of scattered dead wood at stand level, piles of dead wood might be less important for arthropods [42]. The five forest stands in our study varied in amount of dead wood on the forest floor (S1 Table). Two stands had stems left on the ground, while another stand had dead wood piles consisting of branches (similar to those created for our study) and two stands had almost no dead wood on the ground. These differences might have masked effects of the dead wood piles in stands with less dead wood. To conclude, our results align with previous studies in suggesting that dead wood may not always be of importance for arthropod diversity and abundance, and that it is factors such as time and spatial arrangement of the wood that are determining its importance.

#### Predation pressure

The presence of dead wood did not affect the predation pressure of tree-dwelling larvae. We hypothesised that an increase in predator abundance and diversity would have subsequent effects on predation rates. Since we did not detect any differences in abundance or diversity of predators between the dead wood treatments, it is not very surprising that we do not see any differences in attack rates. There was a negative effect of sticky traps on predation rates on tree-dwelling larvae, showing that predators walking up on into the tree on the stem contributes to predation within trees. Even though dead wood addition did not increase predation rates by arthropods on tree-dwelling larvae, there was no decrease either and thus the positive effect of dead wood on cocoon predation by small mammals [4] will remain if dead wood remains present.

#### Implications for pest control and forest management

Our study in combination with studies investigating similar relationship shows foremost that the relationship between the presence of dead wood, arthropod diversity and predation pressure on herbivorous insects is rather complex. We did not find a clear effect of dead wood in our study, however that does not mean that structural diversity, provided by dead wood among others, can be important for both biodiversity [1] and pest control [4,17]. Three aspects that can be attributed to that complexity can be inferred from this study: (i) dead wood could have dissimilar effects on different predator groups [15,16], (ii) the nutrient status and structure of dead wood changes over time with might result in temporal effects on associated communities [4,15], and (iii) dead wood may affect predation rates on different life stages of the pest insect differently [4]. This underlines the need to study the effects of structures such as dead wood during different parts of a pest insect’s life cycle and over time, and to be cautious when generalising on predation effects.

## Supporting information

S1 Table The approximate amount of dead wood on the forest floor (outside the experimental plots) per site. (DOCX)

S2 Table The total number of ground-dwelling arthropods sampled with pitfall traps on plots with and without dead wood in managed pine forest stands. (DOCX)

S3 Table Anova (type II test) and summary table for generalised linear mixed models testing the different response variables (a) total arthropod abundance, (b) total predator abundance, (c) ant abundance, (d) hunting spider abundance and (e) ground beetle abundance in relation to dead wood treatment (added or removed). Dead wood treatment was used as a fixed factor and site as random factor.
(DOCX)

S4 Table Anova (type II test) and summary table for generalised linear mixed models testing the different in (a) number of orders of all arthropods, (b) number of predators groups, c) number of beetle families, d) number of spider families in relation to dead wood treatment (added or removed) Dead wood treatment was used as a fixed factor and site as a random factor.
(DOCX)

S5 Table Anova (type III test) and summary table for generalised linear mixed models testing the difference in proportion of attacks against the dead wood treatment (added or removed). Dead wood treatment, sticky traps and the interaction between dead wood treatment and sticky traps was used as a fixed factors, site and plot nested in site as a random factor.
(DOCX)

S1 Data (XLSX)
