# Supplementary material for: Effects of retained dead wood on predation pressure on herbivores in young pine forests
Source: PLoS One. 2022 Sep 6;17(9):e0273741. doi: 10.1371/journal.pone.0273741 (PMC9447874; doi:10.1371/journal.pone.0273741)
Supplement: S1 Table — (DOCX) [file pone.0273741.s001.docx]

**Table S1.**The approximate amount of dead wood on the forest floor (outside the experimental plots) per site.

| **Site** | **Approx. amount of dead wood on forest floor** |
| --- | --- |
| 3 | Stems on the ground from clearing |
| 5 | A lot of dead wood piles scattered throughout the stand |
| 13 | Stems on the ground from clearing |
| 15 | Almost nothing except from the created piles |
| 17 | Almost nothing except from the created piles |
