# Supplementary material for: Effects of retained dead wood on predation pressure on herbivores in young pine forests
Source: PLoS One. 2022 Sep 6;17(9):e0273741. doi: 10.1371/journal.pone.0273741 (PMC9447874; doi:10.1371/journal.pone.0273741)
Supplement: S2 Table — (DOCX) [file pone.0273741.s002.docx]

**Table S2.**The total number of ground-dwelling arthropods sampled with pitfall traps on plots with and without dead wood in managed pine forest stands.

| Order | Family | Total |
| --- | --- | --- |
| Araneae |  | 2769 |
|  | Agelenidae | 2 |
|  | Araneidae | 1 |
|  | Gnaphosidae | 338 |
|  | Hahniidae | 6 |
|  | Linyphiidae | 1297 |
|  | Lycosidae | 1037 |
|  | Pisauridae | 1 |
|  | Salticidae | 27 |
|  | Tetragnathidae | 1 |
|  | Thomisidae | 30 |
|  | Zoridae | 29 |
| Acari |  | 2929 |
| Coleoptera |  | 970 |
|  | Anthicidae | 1 |
|  | Byrrhidae | 2 |
|  | Cantharidae | 2 |
|  | Carabidae | 74 |
|  | Chrysomelidae | 32 |
|  | Coccinellidae | 1 |
|  | Corticariidae | 3 |
|  | Cryptophagidae | 2 |
|  | Curculionidae | 556 |
|  | Elateridae | 9 |
|  | Leiodidae | 7 |
|  | Lymexylidae | 1 |
|  | Pselaphidae | 1 |
|  | Ptinidae | 1 |
|  | Scarabaeidae | 32 |
|  | Scirtidae | 2 |
|  | Scolytidae | 37 |
|  | Scydmaenidae | 26 |
|  | Silvanidae | 2 |
|  | Staphylinidae | 156 |
|  | Unknown | 23 |
| Hymenoptera |  | 4902 |
|  | Formicidae | 4777 |
| Collembola |  | 2641 |
| Diptera |  | 1040 |
| Heteroptera |  | 84 |
| Homoptera |  | 41 |
| Dermaptera |  | 1 |
| Diplopoda |  | 56 |
| Chiliopoda |  | 8 |
| Orthoptera |  | 1 |
| Blattaria |  | 24 |
| Lepidoptera |  | 9 |
| Psocoptera |  | 32 |
| Isopoda |  | 9 |
|  |  |  |
