# Supplementary material for: Effects of retained dead wood on predation pressure on herbivores in young pine forests
Source: PLoS One. 2022 Sep 6;17(9):e0273741. doi: 10.1371/journal.pone.0273741 (PMC9447874; doi:10.1371/journal.pone.0273741)
Supplement: S3 Table — Anova (type II test) and summary table for generalised linear mixed models testing the different response variables (a) total arthropod abundance, (b) total predator abundance, (c) ant abundance, (d) hunting spider abundance and (e) ground beetle abundance in relation to dead wood treatment (added or removed). Dead wood treatment was used as a fixed factor and site as random factor. (DOCX) [file pone.0273741.s003.docx]

**Table S3**. Anova (type II test) and summary table for generalised linear mixed models testing the different response variables (a) total arthropod abundance, (b) total predator abundance, (c) ant abundance, (d) hunting spider abundance and (e) ground beetle abundance in relation to dead wood treatment (added or removed). Dead wood treatment was used as a fixed factor and site as random factor.

| (a) Total arthropod abundance |  |  |  |  |  |
| --- | --- | --- | --- | --- | --- |
| **Fixed** | Estimates | SE | χ^2^ | df | p-value |
| Intercept | 5.82 | 0.12 |  |  | <0.001 |
| Treatment |  |  | 3.46 | 1 | 0.06 |
| Wood (added) | -0.22 | 0.12 |  |  |  |
| **Random** | Variance | Standard dev. |  |  |  |
| Site | 0.04 | 0.19 |  |  |  |
| **(b) Total predator abundance** |  |  |  |  |  |
| **Fixed** | Estimates | SE | χ^2^ | df | p-value |
| Intercept | 4.91 | 0.17 |  |  | <0.001 |
| Treatment |  |  | 1.69 | 1 | 0.19 |
| Wood (added) | -0.24 | 0.18 |  |  |  |
| **Random** | Variance | Standard dev. |  |  |  |
| Site | 0.06 | 0.24 |  |  |  |
| **(c) Ant abundance** |  |  |  |  |  |
| **Fixed** | Estimates | SE | χ^2^ | df | p-value |
| Intercept | 4.70 | 0.19 |  |  | <0.001 |
| Treatment |  |  | 1.26 | 1 | 0.26 |
| Wood (added) | -0.30 | 0.27 |  |  |  |
| **Random** | Variance | Standard dev. |  |  |  |
| Site | 0.000 | 0.000 |  |  |  |
| **(d) Hunting spiders abundance** |  |  |  |  |  |
| **Fixed** | Estimates | SE | χ^2^ | df | p-value |
| Intercept | 3.33 | 0.14 |  |  | <0.001 |
| Treatment |  |  | 0.02 | 1 | 0.88 |
| Wood (added) | 0.01 | 0.09 |  |  |  |
| **Random** | Variance | Standard dev. |  |  |  |
| Site | 0.09 | 0.29 |  |  |  |
| **(e) Ground beetle abundance** |  |  |  |  |  |
| **Fixed** | Estimates | SE | χ^2^ | df | p-value |
| Intercept | 0.27 | 0.25 |  |  | 0.27 |
| Treatment |  |  | 0.55 | 1 | 0.46 |
| Wood (added) | 0.26 | 0.35 |  |  |  |
| **Random** | Variance | Standard dev. |  |  |  |
| Site | 0.000 | 0.000 |  |  |  |
