# Supplementary material for: Effects of retained dead wood on predation pressure on herbivores in young pine forests
Source: PLoS One. 2022 Sep 6;17(9):e0273741. doi: 10.1371/journal.pone.0273741 (PMC9447874; doi:10.1371/journal.pone.0273741)
Supplement: S4 Table — Anova (type II test) and summary table for generalised linear mixed models testing the different in (a) number of orders of all arthropods, (b) number of predators groups, c) number of beetle families, d) number of spider families in relation to dead wood treatment (added or removed) Dead wood treatment was used as a fixed factor and site as a random factor. (DOCX) [file pone.0273741.s004.docx]

**Table S4**. Anova (type II test) and summary table for generalised linear mixed models testing the different in (a) number of orders of all arthropods, (b) number of predators groups, c) number of beetle families, d) number of spider families in relation to dead wood treatment (added or removed) Dead wood treatment was used as a fixed factor and site as a random factor.

| (a) Order diversity |  |  |  |  |  |
| --- | --- | --- | --- | --- | --- |
| **Fixed** | Estimates | SE | χ^2^ | df | p-value |
| Intercept | 2.22 | 0.07 |  |  | < 0.001 |
| Treatment |  |  | 0.52 | 1 | 0.47 |
| Wood (added) | -0.07 | 0.1 |  |  |  |
| **Random** | Variance | Standard dev. |  |  |  |
| Site | 0.000 | 0.000 |  |  |  |
| **(b) Predator diversity** |  |  |  |  |  |
| **Fixed** | Estimates | SE | χ^2^ | df | p-value |
| Intercept | 1.52 | 0.09 |  |  | < 0.001 |
| Treatment |  |  | 0.06 | 1 | 0.81 |
| Wood (added) | -0.03 | 0.13 |  |  |  |
| **Random** | Variance | Standard dev. |  |  |  |
| Site | 0.000 | 0.000 |  |  |  |
| **(c) Beetle family diversity** |  |  |  |  |  |
| **Fixed** | Estimates | SE | χ^2^ | df | p-value |
| Intercept | 1.6 | 0.1 |  |  | < 0.001 |
| Treatment |  |  | 2.0 | 1 | 0.15 |
| Wood (added) | -0.19 | 0.14 |  |  |  |
| **Random** | Variance | Standard dev. |  |  |  |
| Site | 0.006 | 0.08 |  |  |  |
| **(d) Spider family diversity** |  |  |  |  |  |
| **Fixed** | Estimates | SE | χ^2^ | df | p-value |
| Intercept | 1.44 | 0.1 |  |  | < 0.001 |
| Treatment |  |  | 0.07 | 1 | 0.79 |
| Wood (added) | -0.04 | 0.14 |  |  |  |
| **Random** | Variance | Standard dev. |  |  |  |
| Site | 0.000 | 0.000 |  |  |  |
