# Supplementary material for: Effects of retained dead wood on predation pressure on herbivores in young pine forests
Source: PLoS One. 2022 Sep 6;17(9):e0273741. doi: 10.1371/journal.pone.0273741 (PMC9447874; doi:10.1371/journal.pone.0273741)
Supplement: S5 Table — Dead wood treatment, sticky traps and the interaction between dead wood treatment and sticky traps was used as a fixed factors, site and plot nested in site as a random factor. (DOCX) [file pone.0273741.s005.docx]

**Table S5.**Anova (type III test) and summary table for generalised linear mixed models testing the difference in proportion of attacks against the dead wood treatment (added or removed). Dead wood treatment, sticky traps and the interaction between dead wood treatment and sticky traps was used as a fixed factors, site and plot nested in site as a random factor.

| Larvae attacks (proportion) |  |  |  |  |  |
| --- | --- | --- | --- | --- | --- |
| **Fixed** | Estimates | SE | χ^2^ | df | p-value |
| Intercept | -2.31 | 0.48 |  |  | <0.001 |
| Treatment |  |  | 1.71 | 1 | 0.19 |
| Wood (added) | 0.79 | 0.60 |  |  |  |
| Sticky trap |  |  | 1.71 | 1 | 0.19 |
| Sticky trap (yes) | -0.79 | 0.60 |  |  |  |
| Treatment: Sticky trap |  |  | 0.01 | 1 | 0.91 |
| Wood (added): Sticky trap (yes) | 0.09 | 0.76 |  |  |  |
| **Random** | Variance | Standard dev. |  |  |  |
| Site | 0.006 | 0.08 |  |  |  |
| Site/Plot | 1.5 | 1.23 |  |  |  |
